# *N*,*N*′-Bis[(*E*)-quinoxalin-2-ylmethyl­idene]­ethane-1,2-diamine

**DOI:** 10.1107/S1600536809003006

**Published:** 2009-01-31

**Authors:** Digna Varghese, V. Arun, Manju Sebastian, P. Leeju, G. Varsha, K. K. M. Yusuff

**Affiliations:** a Department of Applied Chemistry, Cochin University of Science and Technology, Cochin 682 022, Kerala, India

## Abstract

In the mol­ecule of the title compound, C_20_H_16_N_6_, the central C—C bond lies on a crystallographic inversion centre. The quinoxalidine ring is nearly planar, with a maximum deviation of 0.021 (2) Å from the mean plane. The crystal structure is stabilized by inter­molecular C—H⋯N inter­actions, leading to the formation of a layer-like structure, which extends along the *a* axis.

## Related literature

For the synthesis of the Schiff base, see: Zolezzi *et al.* (1999 ▶). For the properties of Schiff base ligands, see: Gupta & Sutar (2008 ▶); Harmenberg *et al.* (1991 ▶); Mayadevi *et al.* (2003 ▶); Miller *et al.* (1999 ▶); Naylor *et al.* (1993 ▶); Sreekala & Yusuff (1994 ▶); Xavier *et al.* (2004 ▶); Yusuff & Sreekala (1991 ▶). For related structures, see: Habibi *et al.* (2006 ▶); Taylor & Kennard (1982 ▶).
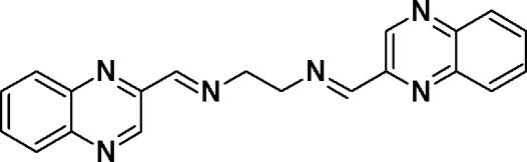


## Experimental

### 

#### Crystal data

C_20_H_16_N_6_
*M* *_r_* = 340.39
Triclinic, 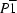

*a* = 6.888 (2) Å
*b* = 7.381 (3) Å
*c* = 9.638 (4) Å
α = 101.674 (6)°
β = 96.233 (6)°
γ = 116.046 (5)°
*V* = 420.1 (3) Å^3^
*Z* = 1
Mo *K*α radiation
μ = 0.09 mm^−1^
*T* = 298 (2) K
0.40 × 0.24 × 0.18 mm

#### Data collection

Bruker SMART CCD area-detector diffractometer
Absorption correction: multi-scan (*SADABS*; Sheldrick, 2001 ▶) *T*_min_ = 0.967, *T*_max_ = 0.995
3956 measured reflections
1465 independent reflections
1239 reflections with *I* > 2σ(*I*)
*R*_int_ = 0.025

#### Refinement

*R*[*F* ^2^ > 2σ(*F* ^2^)] = 0.071
*wR*(*F* ^2^) = 0.164
*S* = 1.27
1465 reflections
118 parameters
H-atom parameters constrained
Δρ_max_ = 0.13 e Å^−3^
Δρ_min_ = −0.21 e Å^−3^

### 

Data collection: *SMART* (Bruker, 2000 ▶); cell refinement: *SAINT* (Bruker, 2000 ▶); data reduction: *SAINT*; program(s) used to solve structure: *SHELXS97* (Sheldrick, 2008 ▶); program(s) used to refine structure: *SHELXL97* (Sheldrick, 2008 ▶); molecular graphics: *SHELXTL* (Sheldrick, 2008 ▶) and/or *ORTEP-3* (Farrugia, 1997 ▶); software used to prepare material for publication: *SHELXL97* and *PLATON* (Spek, 2003 ▶).

## Supplementary Material

Crystal structure: contains datablocks I, global. DOI: 10.1107/S1600536809003006/fj2185sup1.cif

Structure factors: contains datablocks I. DOI: 10.1107/S1600536809003006/fj2185Isup2.hkl

Additional supplementary materials:  crystallographic information; 3D view; checkCIF report

## Figures and Tables

**Table 1:** Hydrogen-bond geometry (Å, °)

*D*—H⋯*A*	*D*—H	H⋯*A*	*D*⋯*A*	*D*—H⋯*A*
C1—H1⋯N2^i^	0.93	2.73	3.647 (4)	168
C9—H9⋯N1^ii^	0.93	2.67	3.593 (3)	169

Symmetry codes: (i) 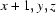; (ii) 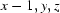.

**Fig. 1.**
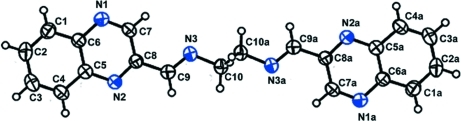
An ORTEP-3 (Farrugia, 1997) plot of the (I) compound, with the atomic labelling scheme. The shapes of the ellipsoids correspond to 50% probability contours of atomic displacement.

**Fig. 2.**
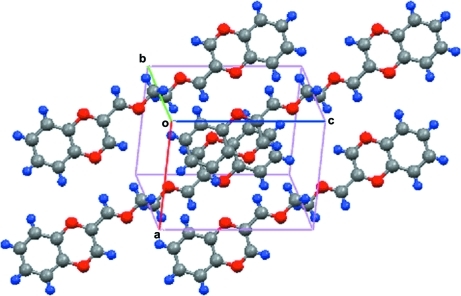
Unit cell packing diagram of N,N'-bis[(E)-quinoxalin-2-ylmethylidene]ethane-1,2-diamine.

**Fig. 3.**
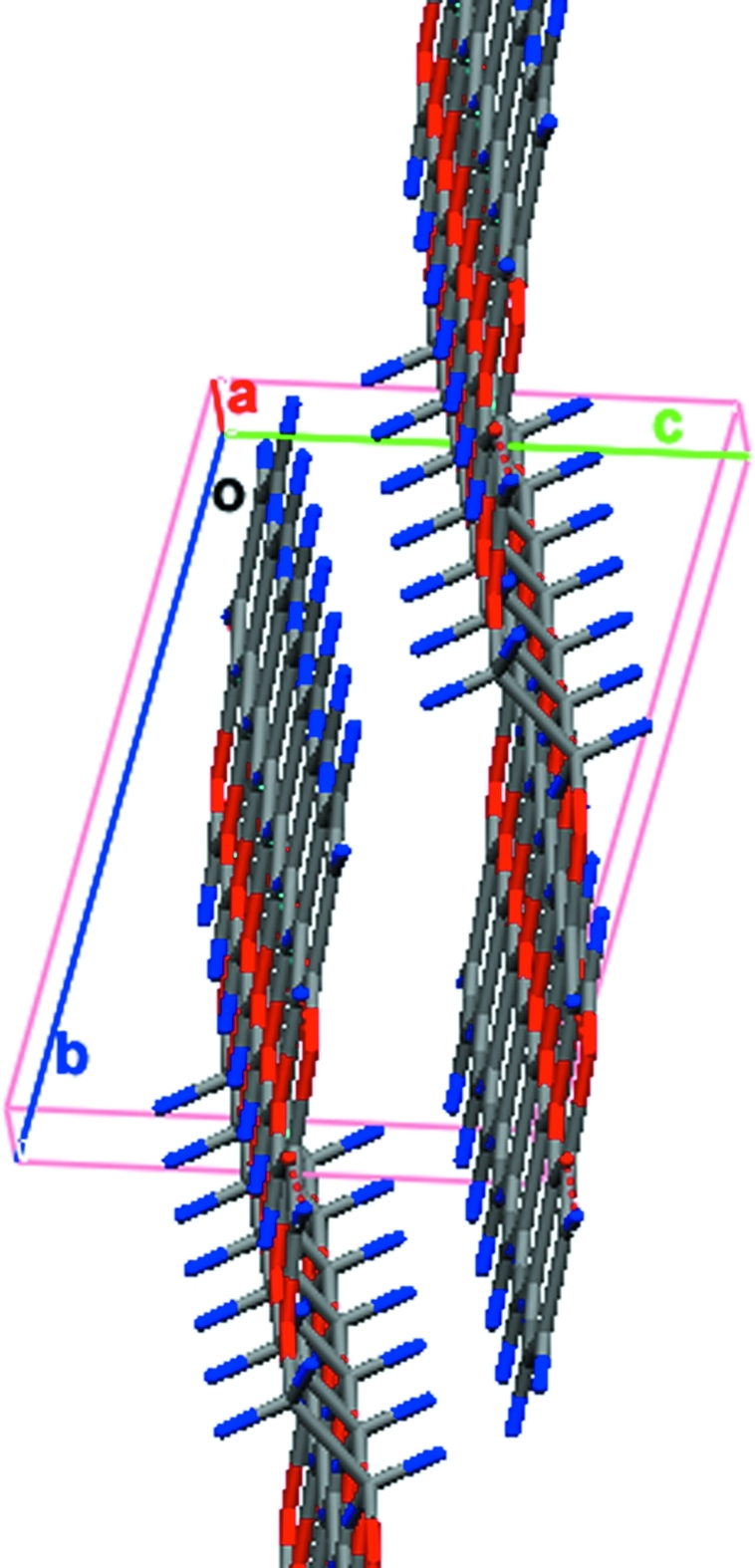
Pairs of (C–)H···N interactions lead to a layer structure along the a axis.

## supplementary crystallographic information

### Comment

Schiff bases derived from aldehydes and diamines constitute one of the most relevant synthetic ligand systems. They find application in a broad range of transition metal catalyzed reactions including lactide polymerization, epoxidation of olefins, hydroxylation and asymmetric ring opening of epoxides (Gupta & Sutar, 2008). Many drug candidates bearing quinoxaline core structures are in clinical trials in antiviral (Harmenberg *et al.*, 1991), anticancer and central nervous system therapeutic areas (Naylor *et al.*, 1993). Catalytic and antibacterial activities have been observed for the Schiff base complexes derived from Quinoxaline-2-carboxaldehyde (Yusuff & Sreekala, 1991; Sreekala & Yusuff, 1994; Mayadevi *et al.*, 2003). Ethylenediamine groups appear to be of importance for various transition metal catalysis (Miller *et al.*, 1999; Xavier *et al.*, 2004). We have recently prepared the title compound (1), and report here its structure.

The single-crystal X-ray structure determination of (1) was carried out at 298 (2) K. The structure analysis showed that the compound to form in triclinic space group P-1 with a =6.888 (2) A°, b=7.381 (3) A°, c=9.638 (4) A° and α = 101.674 (6)°, β = 96.233 (6)°, γ = 116.046 (5)° with *z*=1. A perspective drawing is depicted in figure 1 with the atomic numbering scheme. The C10—N3—C9, N3—C9—C8 angles are 117.9 (2)° and 121.5 (2)° respectively. The N3—C10 and N3—C9 bond lengths are 1.455 (3) A° and 1.260 (3) A° respectively. In this compound (1), the short (C–)H···N contacts are responsible for the stability of layer structure (figure 3) which extends along the *a* axis (Taylor & Kennard, 1982). The (C–)H···N distances and C—H—N angles are given in table 1.

### Experimental

A hot solution of ethylenediamine (1 mmol) in methanol (25 ml) was slowly added over a hot solution of quinoxaline-2-carboxaldehyde (2 mmol) in the same solvent (50 ml). The resulting mixture on cooling yielded the crude product. The precipitated diimine was filtered off and washed with cold methanol. Light yellow single crystals of (1) were obtained from a solution of dichloromethane by slow evaporation.

### Refinement

H atoms were positioned geometrically with, C—H = 0.93 A° and refined in riding mode with *U*_iso_ (H) = 1.2Ueq(C).

### Crystal data

**Table:**

C_20_H_16_N_6_	*Z* = 1
*M**_r_* = 340.39	*F*(000) = 178
Triclinic, *P*1	*D*_x_ = 1.345 Mg m^−^^3^
Hall symbol: -P 1	Mo *K*α radiation, λ = 0.71073 Å
*a* = 6.888 (2) Å	Cell parameters from 1465 reflections
*b* = 7.381 (3) Å	θ = 2.3–25.0°
*c* = 9.638 (4) Å	µ = 0.09 mm^−^^1^
α = 101.674 (6)°	*T* = 298 K
β = 96.233 (6)°	Plate, yellow
γ = 116.046 (5)°	0.40 × 0.24 × 0.18 mm
*V* = 420.1 (3) Å^3^	

### Data collection

**Table:**

Bruker SMART CCD area-detector diffractometer	1465 independent reflections
Radiation source: fine-focus sealed tube	1239 reflections with *I* > 2σ(*I*)
graphite	*R*_int_ = 0.025
φ and ω scans	θ_max_ = 25.0°, θ_min_ = 2.2°
Absorption correction: multi-scan (*SADABS*; Sheldrick, 2001)	*h* = −8→8
*T*_min_ = 0.967, *T*_max_ = 0.995	*k* = −8→8
3956 measured reflections	*l* = −11→11

### Refinement

**Table:**

Refinement on *F*^2^	Primary atom site location: structure-invariant direct methods
Least-squares matrix: full	Secondary atom site location: difference Fourier map
*R*[*F*^2^ > 2σ(*F*^2^)] = 0.071	Hydrogen site location: inferred from neighbouring sites
*wR*(*F*^2^) = 0.164	H-atom parameters constrained
*S* = 1.27	*w* = 1/[σ^2^(*F*_o_^2^) + (0.0542*P*)^2^ + 0.1572*P*] where *P* = (*F*_o_^2^ + 2*F*_c_^2^)/3
1465 reflections	(Δ/σ)_max_ < 0.001
118 parameters	Δρ_max_ = 0.13 e Å^−^^3^
0 restraints	Δρ_min_ = −0.21 e Å^−^^3^

### Special details

**Table:**

Geometry. All e.s.d.'s (except the e.s.d. in the dihedral angle between two l.s. planes) are estimated using the full covariance matrix. The cell e.s.d.'s are taken into account individually in the estimation of e.s.d.'s in distances, angles and torsion angles; correlations between e.s.d.'s in cell parameters are only used when they are defined by crystal symmetry. An approximate (isotropic) treatment of cell e.s.d.'s is used for estimating e.s.d.'s involving l.s. planes.
Refinement. Refinement of *F*^2^ against ALL reflections. The weighted *R*-factor *wR* and goodness of fit *S* are based on *F*^2^, conventional *R*-factors *R* are based on *F*, with *F* set to zero for negative *F*^2^. The threshold expression of *F*^2^ > σ(*F*^2^) is used only for calculating *R*-factors(gt) *etc*. and is not relevant to the choice of reflections for refinement. *R*-factors based on *F*^2^ are statistically about twice as large as those based on *F*, and *R*- factors based on ALL data will be even larger.

### Fractional atomic coordinates and isotropic or equivalent isotropic displacement parameters (Å^2^)

**Table:**

	*x*	*y*	*z*	*U*_iso_*/*U*_eq_	
N1	0.4604 (3)	0.2446 (3)	0.5782 (2)	0.0439 (6)	
N2	0.0673 (3)	0.2571 (3)	0.4735 (2)	0.0405 (6)	
N3	0.0679 (3)	0.3587 (3)	0.8476 (2)	0.0425 (6)	
C1	0.5203 (4)	0.1943 (4)	0.3355 (3)	0.0482 (7)	
H1	0.6488	0.1881	0.3682	0.058*	
C2	0.4561 (5)	0.1772 (4)	0.1921 (3)	0.0518 (8)	
H2	0.5425	0.1608	0.1280	0.062*	
C3	0.2624 (5)	0.1841 (4)	0.1406 (3)	0.0522 (8)	
H3	0.2216	0.1735	0.0429	0.063*	
C4	0.1338 (4)	0.2062 (4)	0.2329 (3)	0.0473 (7)	
H4	0.0032	0.2070	0.1975	0.057*	
C5	0.1963 (4)	0.2279 (4)	0.3820 (3)	0.0374 (6)	
C6	0.3918 (4)	0.2210 (4)	0.4334 (3)	0.0381 (6)	
C7	0.3349 (4)	0.2733 (4)	0.6617 (3)	0.0429 (7)	
H7	0.3764	0.2897	0.7604	0.051*	
C8	0.1382 (4)	0.2813 (4)	0.6116 (3)	0.0371 (6)	
C9	0.0064 (4)	0.3217 (4)	0.7124 (3)	0.0419 (6)	
H9	−0.1251	0.3198	0.6754	0.050*	
C10	−0.0707 (4)	0.3998 (4)	0.9395 (3)	0.0438 (7)	
H10A	−0.1415	0.2835	0.9804	0.053*	
H10B	−0.1857	0.4133	0.8820	0.053*	

### Atomic displacement parameters (Å^2^)

**Table:**

	*U*^11^	*U*^22^	*U*^33^	*U*^12^	*U*^13^	*U*^23^
N1	0.0419 (12)	0.0551 (14)	0.0405 (12)	0.0272 (11)	0.0095 (10)	0.0150 (10)
N2	0.0409 (12)	0.0465 (13)	0.0350 (12)	0.0231 (11)	0.0064 (9)	0.0084 (10)
N3	0.0463 (13)	0.0504 (13)	0.0342 (12)	0.0257 (11)	0.0108 (9)	0.0113 (10)
C1	0.0440 (15)	0.0514 (17)	0.0495 (16)	0.0233 (14)	0.0136 (13)	0.0113 (13)
C2	0.0565 (18)	0.0512 (17)	0.0433 (16)	0.0210 (14)	0.0221 (13)	0.0093 (13)
C3	0.0596 (18)	0.0568 (18)	0.0332 (14)	0.0228 (15)	0.0095 (13)	0.0101 (13)
C4	0.0497 (16)	0.0554 (18)	0.0345 (14)	0.0243 (14)	0.0039 (12)	0.0122 (12)
C5	0.0375 (14)	0.0327 (13)	0.0385 (14)	0.0138 (11)	0.0077 (11)	0.0098 (11)
C6	0.0380 (13)	0.0377 (14)	0.0372 (13)	0.0173 (12)	0.0074 (11)	0.0096 (11)
C7	0.0448 (15)	0.0524 (16)	0.0313 (13)	0.0233 (13)	0.0052 (11)	0.0127 (12)
C8	0.0368 (13)	0.0367 (14)	0.0357 (14)	0.0173 (11)	0.0061 (11)	0.0071 (11)
C9	0.0414 (15)	0.0457 (16)	0.0406 (15)	0.0229 (13)	0.0076 (12)	0.0113 (12)
C10	0.0443 (15)	0.0528 (17)	0.0368 (14)	0.0235 (13)	0.0139 (11)	0.0139 (12)

### Geometric parameters (Å, °)

**Table:**

N1—C7	1.298 (3)	C3—H3	0.9300
N1—C6	1.373 (3)	C4—C5	1.410 (3)
N2—C8	1.315 (3)	C4—H4	0.9300
N2—C5	1.369 (3)	C5—C6	1.409 (3)
N3—C9	1.260 (3)	C7—C8	1.418 (3)
N3—C10	1.455 (3)	C7—H7	0.9300
C1—C2	1.367 (4)	C8—C9	1.472 (3)
C1—C6	1.404 (3)	C9—H9	0.9300
C1—H1	0.9300	C10—C10^i^	1.512 (5)
C2—C3	1.398 (4)	C10—H10A	0.9700
C2—H2	0.9300	C10—H10B	0.9700
C3—C4	1.357 (4)		
			
C7—N1—C6	115.8 (2)	N1—C6—C1	119.7 (2)
C8—N2—C5	116.2 (2)	N1—C6—C5	120.9 (2)
C9—N3—C10	117.9 (2)	C1—C6—C5	119.4 (2)
C2—C1—C6	119.9 (3)	N1—C7—C8	124.0 (2)
C2—C1—H1	120.1	N1—C7—H7	118.0
C6—C1—H1	120.1	C8—C7—H7	118.0
C1—C2—C3	121.0 (3)	N2—C8—C7	121.6 (2)
C1—C2—H2	119.5	N2—C8—C9	116.9 (2)
C3—C2—H2	119.5	C7—C8—C9	121.5 (2)
C4—C3—C2	120.1 (3)	N3—C9—C8	121.5 (2)
C4—C3—H3	120.0	N3—C9—H9	119.3
C2—C3—H3	120.0	C8—C9—H9	119.3
C3—C4—C5	120.7 (3)	N3—C10—C10	109.5 (3)
C3—C4—H4	119.7	N3—C10—H10A	109.8
C5—C4—H4	119.7	C10—C10—H10A	109.8
N2—C5—C6	121.6 (2)	N3—C10—H10B	109.8
N2—C5—C4	119.4 (2)	C10—C10—H10B	109.8
C6—C5—C4	119.0 (2)	H10A—C10—H10B	108.2

Symmetry codes: (i) −*x*, −*y*+1, −*z*+2.

### Hydrogen-bond geometry (Å, °)

**Table:**

*D*—H···*A*	*D*—H	H···*A*	*D*···*A*	*D*—H···*A*
C1—H1···N2^ii^	0.93	2.73	3.647 (4)	168
C9—H9···N1^iii^	0.93	2.67	3.593 (3)	169

Symmetry codes: (ii) *x*+1, *y*, *z*; (iii) *x*−1, *y*, *z*.

## References

[bb1] Bruker (2000). *SMART* and *SAINT* Bruker AXS Inc., Madison, Wisconsin, USA.

[bb2] Farrugia, L. J. (1997). *J. Appl. Cryst.* **30**, 565.

[bb3] Gupta, K. C. & Sutar, A. K. (2008). *Coord. Chem. Rev.* **252**, 1420–1450.

[bb4] Habibi, M. H., Montazerozohori, M., Lalegani, A., Harrington, R. W. & Clegg, W. (2006). *J. Fluorine Chem.* **127**, 769–773.

[bb5] Harmenberg, J., Akesson-Johansson, A., Graslund, A., Malmfors, T., Bergman, J., Wahren, B., Akerfeldt, S., Lundblad, L. & Cox, S. (1991). *Antiviral Res.* **15**, 193–204. 10.1016/0166-3542(91)90066-z 1653556

[bb6] Mayadevi, S., Prasad, P. G. & Yusuff, K. K. M. (2003). *Synth. React. Inorg. Met. Org. Chem.* **33**, 481–496.

[bb7] Miller, J. K., Baag, J. H. & Abu-Omar, M. M. (1999). *Inorg. Chem.* **38**, 4510–4514. 10.1021/ic981450j 11671164

[bb8] Naylor, M. A., Stephen, M. A., Nolan, J., Sutton, B., Tocher, J. H., Fielden, E. M., Adams, G. E. & Strafford, I. J. (1993). *Anticancer Drug. Des.* **8**, 439–461. 8286012

[bb9] Sheldrick, G. M. (2001). *SADABS* University of Göttingen, Germany.

[bb10] Sheldrick, G. M. (2008). *Acta Cryst.* A**64**, 112–122. 10.1107/S0108767307043930 18156677

[bb11] Spek, A. L. (2003). *J. Appl. Cryst.* **36**, 7–13.

[bb12] Sreekala, R. & Yusuff, K. K. M. (1994). *Synth. React. Inorg. Met. Org. Chem.* **24**, 1773–1788.

[bb13] Taylor, R. & Kennard, O. (1982). *J. Am. Chem. Soc.* **104**, 5063–5070.

[bb14] Xavier, K. O., Chacko, J. & Yusuff, K. K. M. (2004). *Appl. Catal. A Gen.* **258**, 251–259.

[bb15] Yusuff, K. K. M. & Sreekala, R. (1991). *Synth. React. Inorg. Met. Org. Chem.* **21**, 553–568.

[bb16] Zolezzi, S., Decinti, A. & Spodine, E. (1999). *Polyhedron*, **18**, 897–904.

